# Clinical characteristics and surgical outcomes in patients with superior oblique muscle palsy: a retrospective study on 1057 patients

**DOI:** 10.1186/s12886-024-03514-6

**Published:** 2024-06-12

**Authors:** Babak Masoomian, Mohammad Reza Akbari, Marrwan Hisham Mohammed, Motahhareh Sadeghi, Arash Mirmohammadsadeghi, Masoud Aghsaei Fard, Masoud Khorrami-Nejad

**Affiliations:** 1 Translational ophthalmology Research center, Farabi Eye Hospital, Tehran University of Medical Sciences, Kargar St, Tehran, Iran; 2 School of Rehabilitation, Tehran University of Medical Sciences, Tehran, Iran; 3 Department of optical techniques, Al-Mustaqbal University College, Hillah, Babylon, 51001 Iraq

**Keywords:** Superior oblique palsy, Abnormal head posture, Clinical characteristics, Strabismus

## Abstract

**Background:**

To evaluate the clinical findings of patients with SOP who underwent surgery.

**Methods:**

This historical cohort study was performed on 1057 SOP patients managed with surgery in Farabi Hospital, Iran, from 2011 to 2022.

**Results:**

There were 990 (93.7%) patients with unilateral SOP with the mean age of 21.8 ± 14.8 years. Of these, 715 patients (72.2%) were diagnosed with congenital SOP, and 275 patients (27.8%) had acquired SOP (*P* < 0.001). In contrast, 67 (6.3%) patients were diagnosed with bilateral SOP, with the mean age of 19.4 ± 15.6 years. Among these, 18 cases exhibited the masked type. The mean angle of vertical deviation in primary position at far in unilateral and bilateral cases was 15.6 ± 8.3 and 13.3 ± 9.1 △, respectively (*P* < 0.001). In unilateral cases, abnormal head posture (AHP) was detected in 847 (85.5%) patients and 12 (1.2%) had paradoxical AHP. Amblyopia was found in 89 (9.9%) unilateral and 7 (10.3%) bilateral cases. Solitary inferior oblique myectomy, was the most common surgery in both unilateral (*n* = 756, 77.1%) and bilateral (*n* = 35, 52.2%) patients. The second surgery was performed for 84 (8.6%) unilateral and 33 (49.3%) bilateral cases (*P* < 0.001). The prevalence of amblyopia and the mean angle of horizontal deviation were significantly higher in patients who needed more than one surgery (all *P* < 0.05).

**Conclusion:**

Congenital SOP was more than twice as frequent as acquired SOP and about 90% of unilateral and 50% of bilateral cases were managed with one surgery. Amblyopia and significant horizontal deviation were the most important factors for reoperation.

**Trial registration:**

The Institutional Review Board approval was obtained from the Tehran University of Medical Sciences (IR.TUMS.FNM.REC.1400.012) and this study adhered to the tenets of the Declaration of Helsinki and HIPAA.

## Background

Superior oblique muscle palsy (SOP) is the most common type of cyclovertical muscle palsy encountered by the ophthalmologist [1–3]. The annual incidence rate was reported 5.73 per 100,000 per year in the United States [3]. 

Superior oblique palsy can be manifested as congenital or acquired. The latter usually happens following closed head trauma or less commonly secondary to respiratory viral infections, central nervous system lesions, and vascular abnormalities, or microvascular ischemia [3] The same clinical features can result from a congenital laxity, attenuated or even absent SO tendon, a developmental abnormality. Sometimes it happens due to unusual pathways of the muscle or functional consequences of extraocular muscle pulleys [1, 4]. 

Congenital SOP has several characteristic signs and symptoms that distinguish it from the acquired type [1, 3]. The most important of these are the early onset and absence of a specific inciting event. Other clinical findings suggestive of congenital SOP include long-standing abnormal head posture, facial asymmetry, amblyopia, and large vertical fusional amplitude [1, 4, 5]. 

Examination of versions usually reveals underaction of the involved superior oblique muscle and over elevation in adduction of its antagonist inferior oblique muscle. However, the function of the superior oblique muscle sometimes can appear normal [6]. 

The common symptoms in patients with SOP are headache, double vision (especially in lateral gazes), and difficulty in reading. Abnormal head postures (head tilt and face turn) can help patients to get rid of diplopia [4]. Facial asymmetry and large vertical fusional amplitudes indicate chronicity [7]. A review of old photography to verify chronicity can avoid unnecessary neurologic evaluation [8, 9]. Surgical management of SOP is challenging, and various surgical methods have been described to tackle SOP, which can involve operating on the ipsilateral oblique muscles, as well as ipsilateral and/or contralateral vertical rectus muscles [10, 11]. 

In this study, we explore the presenting symptoms, clinical features, and management of 1057 consecutive patients with superior oblique palsy who were examined and treated in the strabismus clinic of Farabi eye hospital. To the best of authors’ knowledge, the present study has the largest sample size among previously published articles about patients with superior oblique palsy.

## Methods

This retrospective study was performed based on hospital records of 1057 SOP patients who were managed with surgery at Farabi Eye Hospital, Tehran University of Medical Sciences, from January 2011 to September 2021.

Records of all patients with unilateral or bilateral SOP who underwent strabismus surgery were included. Patients with cerebral palsy, delayed maturation, other coexisting ocular motor nerve palsies, previous ocular surgeries, previous strabismus surgeries and patients with incomplete data recorded in their medical documentation were excluded.

All patients underwent a complete ophthalmic examination and collected data included sex, age, past medical history, presence or absence of remarkable head trauma, laterality of superior oblique muscles palsy (unilateral or bilateral), presence of diplopia, types of abnormal head posture, visual acuity, refractive error, presence of amblyopia, presence of extraocular muscle abnormalities and type of surgeries were collected.

Visual acuity based on the logMAR unit was documented at a 6-meter distance. Dry and cycloplegic refraction were measured by Topcon KR-800 auto-refractometer, and the findings were confirmed by performing retinoscopy. Anisometropia was considered when more than + 1.00 D difference (either sphere or cylinder) existed between two eyes. In amblyopic eyes, VA of 20/40 or worse with an interocular difference of more than two lines was required for enrollment of patients in the unilateral amblyopic group [12]. 

Overaction and underaction of extraocular muscles were assessed by testing ductions and versions in all diagnostic positions of gaze. Oblique muscle dysfunction was graded on a scale from 0 to − 4 for SO underaction and from 0 to + 4 for IO overaction [13]. The Bielschowsky three-step test was performed for all patients. Primary IO overaction was differentiated from bilateral SOP. Bilateral SOP often presents with significant excyclotropia, which are typically absent in IOOA. The Bielschowsky head tilt test is positive in bilateral SOP, showing greater hypertropia in the head tilt position towards the side of the SOP, whereas primary IO overaction usually does not elicit a positive head tilt test. In primary IOOA, over-elevation in adduction is typically more pronounced than in bilateral SOP. Additionally, a compensatory head posture is more common in bilateral SOP than in IOOA. Finally, through the Bielschowsky three-step test, superior oblique muscle palsy was diagnosed with hypertropia in the primary position that increased in ipsilateral head tilt compared to contralateral tilt [3, 5, 13]. In young children, to confirm SOP, short-time unilateral patching was performed to look for correction of the head posture after patching.

The angle of deviation was measured using an alternate prism cover test with the prism placed in front of the paretic eye in six gazes. Gazes included: (1) distance of 6 m in primary position, (2) near at 33 centimeters, (3) ipsilateral gaze, (4) contralateral gaze, (5) ipsilateral head tilt, and (6) contralateral head tilt of 30 degrees to each side.

We used the following definitions in our study:

1. Unilateral SOP: This term was defined for patients with incomitant hypertropia, which increases on the contralateral gaze. The patients had positive Bielschowsky’s head-tilt test with unilateral SO underaction and /or inferior oblique (IO) overaction.
2. Bilateral SOP: These patients had both SO muscles underaction and/or overaction of both IO muscles in version test; associated with positive Bielschowsky’s head-tilt test on either side. Inversion of hypertropia in one of the nine gaze positions can be an indicator of Bilateral SOP. This condition is characterized by hypertropia that inverts from one eye to the other eye when the patient’s gaze is changed from looking one way to looking the opposite way [13]. Diagnosis of masked bilateral superior oblique palsy is established when signs of SOP appear in the normal eye of a patient who has undergone strabismus surgery for SOP in the contralateral eye [1, 13].
3. Congenital SOP: was considered in patients with an onset of vertical strabismus or any abnormal head posture since early childhood either by history or old family photo albums, absence of diplopia, a large range of vertical fusion vergence or presence of commitancy in vertical deviation. Patients may have a combination of these criteria [14, 15].
4. Acquired SOP: These patients had a positive history of head trauma secondary to fall, car or motor accidents, any orbital/intracranial surgery, assault or ischemic etiology (e.g., hypertension and diabetic mellitus), which was considered significant enough to induce SOP [15].

Primary inferior oblique overaction was differentiated from unilateral superior oblique palsy by the absence of head posture, no hypertropia in the primary position no superior oblique underaction on version testing and finally, through the Bielschowsky three-step test which in SOP, the hypertropia in the affected eye was increased in ipsilateral head tilt [16, 17]. 

The choice of surgical procedure was primarily based on the amount of vertical deviation in the primary position and the specific clinical presentation of each patient. Simple IO myectomy was preferred as the initial procedure, particularly in patients with overaction of the ipsilateral IO and preoperative hyperdeviation of 10 to 15 PD or slightly more, without significant SO tendon laxity as determined by the torsional force duction test during surgery [12, 18–21]. 

Inferior rectus (IR) recession was indicated in cases where significant hypertropia could not be managed by IO myectomy alone. This procedure was chosen either alone on the non-paretic side or in combination with IO myectomy, particularly when the paretic eye was the dominant eye for fixation [22]. 

The superior rectus (SR) muscle was unlikely to be chosen as the sole muscle for surgery. Instead, it was often selected as a secondary muscle in cases of vertical deviation greater than 15 PD in the primary position, especially in the presence of spread of commitancy and SR muscle contracture. Complete deviometry had to be performed in different gazes to determine the appropriate surgical approach [23]. 

SO tuck was indicated in cases of significant SO underaction, chosen to strengthen the paretic SO muscle. Isolated SO tucking was appropriate for patients with unilateral SOP who had small hypertropia (some believed less than 10 PD) in the primary position, which was incomitant and associated with significant underdepression in adduction on the affected side, with minimal to no overelevation in adduction. The presence of a floppy SO tendon in the duction stress test and excyclotorsion equal to or more than 5 degrees were important considerations. SO tucking combined with surgery on the vertical recti (either the ipsilateral SR or contralateral IR) could be performed in patients with larger deviations [24]. 

The data was analyzed using SPSS-version 24 software (IBM, Armonk, NY, USA). Normal data distribution was tested by Shapiro–Wilk and according to the normal distribution of the data, two-independent sample t-test was applied to determine statistically significant differences in some variables between congenital and acquired groups. Mann-Whitney test was applied to compare quantitative variables between congenital and acquired groups. χ2 was also used to evaluate the significant differences in qualitative variables such as gender and laterality in each group. The Wilcoxon test was performed to compare the parameters between the eye with superior oblique palsy and the normal eye and the mean angle of deviation in different gazes in patients with unilateral superior oblique palsy. *P*-values less than 0.05 are considered significant.

## Result

This historical cohort study was performed on 1057 patients with SOP who underwent surgery, including 557(52.7%) males and 500 (47.3%) females (*P* = 0.08) with a mean age of 21.7 ± 14.8 (range, 1–82) years. Unilateral SOP was observed in 990 (93.7%) patients, and 67 (6.4%) patients had bilateral involvement (*P* < 0.001).

Congenital and acquired etiologies were found in 764 (72.3%) and 293 (27.7%) patients, respectively (*P* < 0.001). The mean age of patients at the time of surgery was 17.4 ± 12.1 (range 1–58) years for congenital and 32.6 ± 15.6 (range 3–82) years for the acquired SOP group (*P* < 0.001).

Of 957 patients who had cooperation for measuring CVDA, the prevalence of amblyopia was 10% (*n* = 96) which was mild in 72 (75%), moderate in 17 (18%) and severe in 7 (7%) patients. The Mean preoperative vertical deviation in primary position was 14.6 ± 7.9 (range, 2–45) prism diopter (PD) at near and 15.4 ± 8.2 (range, 0–50) PD at distance. Vertical deviation in bilateral cases was significantly less than in unilateral cases in distance measurement (13.3 ± 9.1 vs. 15.6 ± 8.3 PD, *P* < 0.001).

Of all SOP cases, 940 (88.9%) patients required only one procedure and 117 (11.1%) cases underwent two or more surgeries. Isolated inferior oblique myectomy was the most common first type of surgery in both unilateral (*n* = 756, 77.1%) and bilateral (*n* = 35, 52.2%) groups. Surgery was performed on the paretic superior oblique muscle in 42 (4.0%) patients alone or combined with other vertical muscles. Of 1057 SOP cases, horizontal strabismus (> 10 PD) was observed in 119 (11.3%), where 60 (5.7%) had it addressed in the first surgery, 55 (91%) for exotropia and 5 (9%) for esotropia.

### A. Unilateral Superior Oblique Palsy

Unilateral SOP was found in 990 (93.7%) patients with a mean age of 21.8 ± 14.8 (range, 2–84) years and slightly higher prevalence in men (*n* = 525, 53.6%) and in the right eye (*n* = 518, 52.9%). The congenital and acquired etiologies of SOP in unilateral cases were observed in 715 (72.2%) and 275 (27.8%) patients (*P* < 0.001). The mean age of congenital and acquired unilateral SOP patients at the time of surgery was 17.7 ± 12.1 (range 1–58) and 32.7 ± 15.6 (range 3–82) years, respectively (*P* < 0.001). In the congenital group, 364 (50.9%) were males, and 351 (49.1%) were females (*P* = 0.627), but the frequency for males and females in acquired type was 163 (59.3%) and 112 (40.7%) patients, respectively (*P* = 0.02). In acquire SOP group, the mean duration between the onset of paresis and surgery was 3.8 ± 3.5 years (range, 6 months- 20 years).

Of 898 patients with Unilateral SOP who cooperated for measuring (CDVA), the mean of CDVA in the affected eye and the other eye was 0.03 ± 0.11 and 0.04 ± 0.01 logMAR, respectively (*P* = 0.870). Of these patients, 727 (78.2%) had 0 LogMAR in both eyes, and amblyopia was 9.9% (*n* = 89). The severity of amblyopia was mild in 67(75%), moderate in 15(17%) and severe in 7(8%) cases.

Information on the presence or absence of diplopia was recorded in 855 (272 acquired and 583 congenital) patients over five years old. Diplopia was reported in 148 (17.3%) of all unilateral patients. The frequency of diplopia in acquired cases (*n* = 88, 32.4%) was significantly higher than in congenital patients (*n* = 60, 10.3%) (*P* < 0.001).

In unilateral cases, 847 (85.6%) patients (578 congenital and 269 acquired) had abnormal head posture. Contralateral abnormal head posture was found in 835 (84.3%) patients (574 congenital and 261 acquired), and 12 (1.2%) patients (four congenital and eight acquired) had a paradoxical abnormal head posture. There was no report of AHP in the records of 143 (14.4%).

Remarkable Ipsilateral superior oblique underaction (≤ -2) was detected in 290 (27.4%), and Ipsilateral inferior oblique overaction ( ≥ + 2) was seen in 884 (83.6%) patients. The prevalence of oblique muscles dysfunctions in adduction in all congenital and acquired groups are shown in Table 1. The mean ipsilateral IO overaction in congenital unilateral SOP was significantly higher than acquired ones (2.0 ± 0.7 vs. 1.8 ± 0.7, *P* = 0.02). However, the mean ipsilateral SO underaction in the congenital group did not have a significant difference with acquired unilateral SOP patients (2.3 ± 0.6 vs. 2.2 ± 0.7, *P* = 0.570).

**Table 1:** The grading of ipsilateral overelevation and under depression in adduction in unilateral SOP patients

		Group		
		Congenital *N* = 764	Acquired *N* = 293	Total *N* = 1057
Over elevation in adduction	+ 1	123 (16.1%)	50 (17%)	173 (16.4%)
	+ 2	490 (64.1%)	187 (63.8%)	677 (64.0%)
	+ 3	142 (18.6%)	56 (19.1%)	198 (18.7%)
	+ 4	9 (1.2%)	0 (0.0%)	9 (0.9%)
Under depression in adduction	0	514 (67.3%)	202 (68.9%)	716 (67.7%)
	-1	29 (3.8%)	22 (7.5%)	51(4.8%)
	-2	179 (23.4%)	60 (20.5%)	239 (22.6%)
	-3	42 (5.5%)	9 (3.1%)	51 (4.8%)
	-4	0.0 (0.0%)	0.0 (0.0%)	0 (0.0%)

N, number

The mean hypertropia at distance in the congenital group was 15.6 ± 8.2 (range, 2–50) PD, and in the acquired group was 15.0 ± 8.6 (range, 2–50) PD (*P* = 0.342). At near it was 15.0 ± 7.8 PD (range, 2–50) for the congenital and 13.6 ± 8.0 (range, 2–45) PD for the acquired group (*P* = 0.03). In the unilateral group, 348 (35%) of 990 patients had horizontal deviation of more than 5 (range, 5–85) PD. The mean angle of ocular deviation in the unilateral SOP is shown in Table 2.

**Table 2:** the mean angle of deviation in different gaze positions in patients with unilateral SOP

			Minimum (PD)	Maximum (PD)	Mean (PD)	*P*-value*
Primary position	Vertical deviation	Far	2.0	50.0	15.6 ± 8.2	*P* < 0.001
		Near	2.0	45.0	14.7 ± 7.9	
	Horizontal deviation	Far	0.0	86.0	9.6 ± 6.9	*P* < 0.001
		Near	0.0	88.0	10.3 ± 7.3	
Lateral gazes	Vertical deviation	Ipsilateral	0.0	50.0	10.1 ± 8.2	*P* < 0.001
		Contralateral	2.0	50.0	18.4 ± 9.8	
Head tilt	Vertical deviation	Ipsilateral	2.0	55.0	18.6 ± 9.5	*P* < 0.001
		Contralateral	0.0	45.0	10.9 ± 8.2	

PD, prism diopter

*Wilcoxon Signed Ranks Test

Of 990 unilateral cases, 906(91.5%) patients required only one procedure and 84(8.5%) cases had two or more surgeries. The rate of amblyopia frequency was significantly higher in patients who needed two or more surgeries (*n* = 23, 27.4%) compared to patients who were managed with only one surgery (*n* = 66, 7.3%) (*P* < 0.001).

The most common surgical procedure planned was IO myectomy (*n* = 756, 76.4%) followed by ipsilateral SR recession (*n* = 48, 4.8%), contralateral IR recession (*n* = 25, 2.5%) and ipsilateral IR resection (*n* = 10, 1.0%). Superior oblique tuck was performed for 18 (1.8%) patients. In 176 (17.8%) unilateral cases, surgery was planned for more than one vertical muscle in the first surgery due to a large amount of hypertropia (Table 3). Also, concurrent horizontal muscle surgeries were performed in the first surgery for 53(5.3%) patients. In patients with unilateral SOP, the mean angle of hypertropia in different gaze positions for each surgical plan are reported in Table 4.

**Table 3:** Evaluating the effective factors on the reoperating rate in unilateral SOP patients

		Surgery times	Mean ± SD	Mean Difference	95% Confidence Interval of the Difference		*P*-value*
					Lower	Upper	
Age (year)		One surgery	21.6 ± 14.7	-1.0	-3.8	1.8	0.492
		Two or more surgeries	22.6 ± 15.5				
Vertical deviation (primary position, PD)	Far	One surgery	15.3 ± 8.2	-0.9	-3.0	1.3	0.433
		Two or more surgeries	16.2 ± 9.2				
	Near	One surgery	14.4 ± 7.8	-1.4	-3.5	0.7	0.182
		Two or more surgeries	15.8 ± 8.5				
Horizontal deviation (primary position, PD)	Near	One surgery	10.0 ± 6.3	-3.4	-5.5	-1.2	**0.002**
		Two or more surgeries	13.3 ± 12.4				
	Far	One surgery	9.4 ± 5.9	-2.7	-4.8	-0.5	**0.014**
		Two or more surgeries	12.1 ± 13.0				
CDVA difference between eyes (logMAR)		One surgery	0.03 ± 0.11	-0.057	-0.087	-0.026	**< 0.001**
		Two or more surgeries	0.09 ± 0.16				

* Two-independent sample t-test

PD: prism diopter; CDVA: best-corrected distance visual acuity

**Table 4:** The mean angle of vertical deviation in different surgical plans in patients with unilateral superior oblique palsy

	Ipsilateral gaze (PD)	Contralateral head tilt (PD)	Primary position (PD)		Ipsilateral head tilt (PD)	Contralateral gaze (PD)
			Near	Far		
Isolated IO myectomy	9.8 ± 9.4	10.1 ± 7.3	13.9 ± 7.3	14.7 ± 7.5	18.7 ± 10.4	17.7 ± 9.3
IO myectomy plus ipsilateral SR recession	16.0 ± 11.1	19.4 ± 13.75	20.1 ± 12.4	21.3 ± 13.7	26.6 ± 16.05	26.96 ± 11.2
IO myectomy plus Contralateral IR recession	9.1 ± 11.2	9.6 ± 8.0	14.5 ± 7.5	15.2 ± 8.6	15.2 ± 10.6	16.3 ± 14.5
IO myectomy plus SO tuck	8.0 ± 2.5	11.0 ± 6.6	12.5 ± 9.0	13.1 ± 10.1	15.3 ± 11.4	22.0 ± 18.2

IO: inferior oblique, SR: superior rectus, SO: superior oblique, IR: inferior rectus

Comparisons between patients who were managed with one surgery and those needed two or more surgeries are shown in Table 4. As shown in this table, the mean angle of horizontal deviation at near and distance was significantly lower in cases who were managed with only one surgery (*P* = 0.002 and *P* = 0.014, respectively). Also, the CDVA difference between the two eyes was significantly higher in cases who needed more than one surgery (< 0.001).

### B. bilateral Superior Oblique Palsy

Of 1057 patients, 67 (6.3%) cases had bilateral SOP, including 30 (45%) males and 37 (55%) females with a mean age of 19.4 ± 15.6 (range, 1–73) years. The congenital and acquired causes for bilateral cases were observed in 49 (73.1%) and 18 (26.9%) patients, respectively (*P* < 0.001). The mean age of patients with congenital and acquired bilateral SOP at the time of surgery was 12.4 ± 11.3 (range, 1–58) and 30.3 ± 8.0 (range, 22–53) years, respectively (*P* < 0.001). Eighteen bilateral cases (26.9%), comprising 14 congenital and 4 acquired patients, were diagnosed as masked bilateral SOP after the first surgery, when hypertropia manifested in the contralateral eye. The mean CDVA in the right and left eye was 0.04 ± 0.11 (range 0.0–0.7) and 0.03 ± 0.08 (range 0.0–0.4) logMAR, respectively. In bilateral cases, amblyopia was detected in 7(10.4%) patients. Information on the presence or absence of diplopia had been recorded in 53 (18 acquired and 35 congenital) patients above five years of age. Diplopia was reported in 16 (23.9%) of all bilateral patients. The frequency of diplopia in acquired cases (*n* = 10, 55.5%) was significantly higher than in congenital patients (*n* = 6, 17.1%) (*P* < 0.001). In this group, 58 (86.6%) patients had head tilt.

The mean angle of hypertropia at distance and near was 14.3 ± 9.1 (range 2–40) PD and 13.4 ± 8.3 (range 2–35) PD, respectively. Concurrent horizontal deviation (> 5 PD) was detected in 14(20.9%) of patients, 12.5 ± 10.4 (range 0–40) PD at near and 13.4 ± 8.3 (range 0–30) PD at distance. There were no significant differences between the mean amount of hypertropia in the right (12.9 ± 9.2 PD) and left (13.6 ± 9.8 PD) gazes (*P* = 0.66), as well as between right (14.5 ± 7.3 SD) and left (10.0 ± 6.5 PD) head tilts (*P* = 0.06).

Of the patients with bilateral SOP in this study, the most frequently performed procedure was IO myectomy, which was done in 35 (52.2%) patients. The second most common procedure was bilateral IR recession, performed in 12 (17.9%) cases, and then the Harada-Ito procedure was done in 4 (6.0%) patients. Out of the total 67 bilateral SOP cases, 34 (51%) patients required only a single surgical procedure, while 33 (49%) patients needed two or more surgeries.

## Discussion

The current study was to evaluate the clinical characteristics of 1057 subjects with SOP. Except for the study by Heo et al. [18], other published articles regarding SOP have a significantly small sample size compared to the present study. Albeit Heo et al [25] study was conducted using claims data that excluded the patients with acquired SOP and did not provide information on the angle of deviation, presence of abnormal head posture or visual acuity. Compared to previous studies, the higher number of patients in this study allows us to report the results of data analysis with greater accuracy.

The clinical manifestations of SOP have been reviewed in a few studies [5, 14, 17]. Like most of them, we observed a predominance of disease in younger patients (mean, 21.7 ± 14.8 years). The mean age of patients in the present study is close to Van Noorden (24.7 years) and Bagheri et al. (19.7 ± 11.7 years) studies [1, 26]. We have observed that the younger age of the patients influences the presentation and surgical management strategy of patients with SOP. Younger patients often require more aggressive interventions due to the severity of the symptoms.

Same as previous studies [26, 27], there was a preference for males (52.7% vs. 47.3%), and compared to acquired causes for SOP, most of the patients were of congenital type (27.7% vs. 72.3%). Ellis et al. study on 108 SOP patients showed that 76.9% of cases were congenital [5], which is almost similar to our finding. Similar to the Ellis study, the congenital group in Bagheri et al. report was 76.7% of the total [26], while in the study by Helveston et al. the congenital group constituted only 43.9% of the total [28]. This remarkable difference can be due to their small sample size (*n* = 82).

We noticed that congenital SOP requiring surgery was twice as frequent as acquired SOP, raising the possibility that acquired SOP may have resolved more frequently, even spontaneously. The question arises whether the presence or absence of diplopia is a helpful symptom in distinguishing between acquired and congenital paralysis.

Diplopia is more commonly reported in patients with recently acquired SOP; however, in the present study, nearly 11% of all congenital cases reported diplopia; therefore, it seems that the presence of diplopia cannot confirm the acquired cause, since patients with decompensated congenital SOP can also report diplopia. This observation guides us to consider diplopia as a significant symptom for both acquired and congenital SOP. Therefore, the management strategy should be tailored considering the presence of diplopia, regardless of the type of SOP.

Bilateral SOP was present in only 6.3% of our cohort. This is comparable with results from Mollan et al., who had 10 (6.6%) of bilateral palsy among 150 cases [17], and slightly less than those in the study of Gunderson et al., who reported 12 (9.6%) bilateral cases among 124 SOP cases [29]. Prevalence of bilateral type should probably be higher than our reported value because most bilateral cases have an acquired origin and recover before surgery. Such prevalence should alert clinicians to the possibility of bilateral SOP in patients with acquired condition and suggest a conservative approach with close monitoring before proceeding to surgery.

In the present study, amblyopia was detected in 9% of all patients who cooperated for visual acuity control, which was close to Holmes et al. study (7.5%) [30]. While the prevalence of amblyopia in Bagheri et al. [26] and Tarczy-Hornoch et al. [31], studies reported 19% and 17%, respectively. Of course, the sample size in the current study is not comparable with the last two studies. Amblyopic patients often have poor or absent stereopsis, which can limit the patient’s ability to achieve binocular single vision postoperatively. This challenge often results in a more complex strabismus that may be less responsive to a single surgical intervention, thus necessitating the need for additional surgeries. Moreover, the presence of amblyopia after surgery often indicates a more severe or longstanding strabismus, making the surgical correction more challenging and increasing the likelihood of needing multiple surgeries. Therefore, patients with amblyopia, compared with patients with normal vision, have a lower chance of obtaining or achieving binocular single vision following surgery. This could be the main reason why in our study, those requiring more surgeries had a higher prevalence of amblyopia compared to patients who were managed with a single surgery. Considering the potential of amblyopia present in SOP patients, a comprehensive visual function assessment should be considered in the diagnostic workup.

A head tilt toward the shoulder of the unparalyzed side is considered as a classic sign of unilateral involvement SOP but, it is not an exclusive sign of SOP and can be seen in other vertical muscles pathology or neurologic pathology [13, 32]. Head tilt was detected in a significant number of patients (85.6%) in the present study. The rate of head tilt in Merino Sanz study was reported 94.5% [33]. The absence of head tilts has been explained by the inability to obtain single binocular vision by any head position, or the presence of large fusional amplitudes or reduced visual acuity in one. Severe amblyopia may be one of the reasons, as was mentioned in Debaji Ray’s study [27], but in the present study, we were unable to reach this conclusion. If the head tilted toward the contralateral eye is not enough for overcoming the residual vertical deviation and diplopia, or if the fusion was only intermittent, some patients prefer to have a head posture that elicits double vision with wide image separation Therefore, some patients use of paradoxical head tilts to disrupt the fusion. The paradoxical head tilt was detected in 12 (1.2%%) of unilateral patients. In Debaji et al. study, 2 of 181 SOP patients had the paradoxical head tilt with posttraumatic SOP with a large vertical deviation of more than 25 PD [27]. Khan et al. reported a 1-year-old baby showing paradoxical diplopia in whom the paralytic eye was the fixating eye [34]. This report shows that paradoxical diplopia can be seen in both congenital and acquired types. This suggests that surgical planning should consider the patient’s head posture and the potential for a paradoxical head tilt.

Some SOP patients present with concomitant horizontal deviation. In Merino Sanz study, 51.3% of patients had horizontal strabismus, mainly exotropia, in which, 6.6% required surgery [33]. Associated horizontal strabismus has been reported in 50–60% of SOP patients, both in congenital and in acquired type [26]. Helveston et al. reported that 36% of SOP cases had concomitant horizontal deviation and 10% of them underwent combined horizontal surgery [12]. Based on some studies, horizontal rectus muscle surgery was mostly needed in horizontal deviations larger than 10 PD, since most of the lower amounts could be resolved after surgery for only cyclovertical muscles [12, 26]. In our report, horizontal strabismus (> 10 PD) was detected in 119 (11.3%) patients, and only 60 (5.7%) of them were managed with appropriate horizontal muscle surgery.

The entity of the masked bilateral SOP has been described by Kushner [35], where the hypertropia of the lesser affected eye, was not present in any field of gaze or in any head tilt. In the Kushner series, 9 of 28 (32%) bilateral cases had significant asymmetric involvement and masked type. Eighteen (26.9%) of 67 bilateral cases in the present study had similar presentation; Therefore, the examiner should be cautious about the possible involvement of the second eye, which may become manifest after surgery on the hypertropic eye [35]. This emphasizes the importance of careful preoperative evaluation to detect masked bilateral SOP and adapt surgical plans accordingly to prevent postoperative surprises.

The various surgical procedures for the management of SOP were planned mostly based on the amount of the vertical deviation at the primary position, and the method chosen by the surgeon to correct the deviation. Some prefer operating on one muscle only for the first procedure to avoid overcorrections and waiting before planning a second intervention [35]. This highlights that the surgical management of SOP should be individualized based on the patient’s specific clinical presentation and the surgeon’s judgment.

Reports from different studies show that surgery for SOP is successful in 58–95% of cases [26, 36, 37]. However, older age, greater vertical and torsional deviations and amblyopia have been mentioned as predisposing factors for reoperation [33, 38, 39]. In the present study, the second surgery was performed for 117 (11.1%) of patients compared to 25% in Sanz et al. study [33]. In the recently published cohort study by Heo et al., the reoperation rate for SOP was 10.7% which is close to our finding. In their study, patients who underwent surgery that included the superior oblique muscle or patients who had surgery on more than one vertical muscle (excluding superior oblique muscle) had increased hazard ratios for reoperation [25]. Significant horizontal deviation, the CDVA difference between eyes and the presence of amblyopia, were factors that increased the chance of needing two or more times surgery in the present study. These findings underline the importance of considering these risk factors in preoperative counseling and surgical planning to manage patient expectations and reduce the need for reoperations.

The retrospective nature of this study caused some limitations, including the unavailability of some data. We did not have reliable data about facial asymmetry or subjective or objective cyclotorsion in our patients. However, this study contains the weaknesses inherent to any retrospective, non-randomized study, including missing data and a lack of standardized treatment plans across all cases. In addition, since our focus was on SOP patients who underwent surgical intervention, we did not utilize the Knapp classification for SOP as proposed by Knapp P [40]. 

## Conclusion

This retrospective study provides significant insights into the clinical characteristics and surgical outcomes of patients with SOP. Our findings highlighted that congenital SOP was more predominant, accounting for nearly 72.2% of unilateral SOP cases. The majority of unilateral (90%) and half of bilateral SOP cases only required a single surgical intervention for management, with solitary inferior oblique myectomy being the most common surgical procedure. Our study underscores the importance of considering amblyopia and significant horizontal deviation as critical factors in determining the need for reoperation. These factors were significantly more prevalent among patients who required more than one surgery. We recommend that clinicians pay particular attention to these factors during the initial diagnosis and treatment planning stages. Early identification and management of these aspects could potentially reduce the need for multiple surgeries and improve the overall prognosis for patients with SOP. Furthermore, the higher prevalence of congenital SOP in our cohort suggests a need for early and routine screening for SOPs in children, especially those presenting with abnormal head postures. Early detection and treatment could enhance outcomes and potentially prevent long-term complications associated with this condition. Our study further signifies the need for personalized patient management strategies, considering the individual patient’s clinical characteristics and the type of SOP. Future research should aim to validate our findings in other populations and investigate the long-term outcomes of different surgical approaches in managing SOP.

## Data Availability

The datasets used and analyzed during the current study are available from the corresponding author on reasonable request.

## Abbreviations

SOP: Superior Oblique muscle Palsy
CDVA: Best-corrected distance visual acuity
IO: Inferior Oblique
AHP: Abnormal Head Posture
